# Toward Standardized Management of Indeterminate Thyroid Nodules in Pediatric Patients: A Systematic Review and Call for a Comprehensive Risk Stratification Model

**DOI:** 10.3390/jcm14176112

**Published:** 2025-08-29

**Authors:** Gerdi Tuli, Jessica Munarin, Anna Biga, Francesco Quaglino, Giulia Carbonaro, Luisa De Sanctis

**Affiliations:** 1 Department of Public Health and Pediatrics, University of Turin, 10126 Turin, Italy; jessica.munarin@unito.it (J.M.); anna.biga@unito.it (A.B.); luisa.desanctis@unito.it (L.D.S.); 2 Department of Pediatric Endocrinology, Regina Margherita Children’s Hospital, 10126 Turin, Italy; 3 Department of General Surgery, Maria Vittoria Hospital, ASL City of Turin, 10154 Turin, Italy; francesco.quaglino@aslcittaditorino.it (F.Q.); giulia.carbonaro@aslcittaditorino.it (G.C.)

**Keywords:** indeterminate nodules, pediatric age, surgical approach, clinical management, children and adolescents

## Abstract

**Background/Objective:** Thyroid nodules are rare in the pediatric population but carry a higher malignancy risk compared to adults. Evaluation and management of cytologically indeterminate nodules vary considerably between institutions and countries. The aim was to systematically review current evidence on the management of indeterminate thyroid nodules in the pediatric population. **Methods:** A systematic review of the literature was conducted, focusing on cytological classification systems, surgical strategies, and the use of ancillary tools such as molecular testing. **Results:** Most studies (42.9%) recommend lobectomy for indeterminate thyroid nodules in children; however, considerable heterogeneity in management strategies was observed among institutions. This variability precluded the possibility of conducting a meta-analysis of surgical outcomes. Additionally, a lack of pediatric-specific risk of malignancy (ROM) data for the British Thyroid Association (BTA) and SIAPEC cytological classification systems was noted. **Conclusions:** We propose the development of a pediatric-specific, multiparametric risk stratification model that incorporates clinical features, biochemical markers, ultrasound characteristics, cytological classification, and molecular profiling. This comprehensive score could help standardize the management of indeterminate thyroid nodules in children and guide clinical decision-making, ranging from observation to total thyroidectomy. Prospective validation in multicenter pediatric cohorts is essential to confirm its clinical utility.

## 1. Introduction

The prevalence of solid thyroid nodules in the pediatric population is estimated to be approximately 1–1.7%, with incidence increasing with age [1]. The growing number of pediatric cancer survivors, combined with the enhanced sensitivity of modern imaging technologies, has contributed to an increase in the incidence of thyroid carcinoma among children and adolescents. Several studies report an average annual increase of around 4% [2,3,4]. Compared to adults, thyroid nodules in children carry a higher risk of malignancy (ROM), with reported rates of 20–25% versus 5–10% in adults [5,6]. Moreover, children with malignant nodules are more likely to present with advanced-stage disease at diagnosis [6]. Nevertheless, the overall prognosis in children remains excellent, with long-term survival rates exceeding 97–99% [7].

The initial evaluation of thyroid nodules in pediatric patients relies on a multimodal diagnostic approach. This includes ultrasonographic assessment, measurement of thyroid function (Thyroid Stimulating Hormone [TSH], free T4, and free T3), and determination of anti-thyroid antibodies (anti-thyroid peroxidase and anti-thyroglobulin, the latter being positive in approximately 20–25% of cases). Radionuclide scintigraphy is indicated in the presence of a thyrotoxic state, while fine-needle aspiration biopsy (FNAB) remains the gold standard for cytological assessment [6].

While the Thyroid Imaging Reporting and Data System (TIRADS) is validated and widely used in adults, its utility in pediatric populations remains limited [8]. Evidence suggests that both EU-TIRADS and ACR-TIRADS may miss up to 25% of pediatric thyroid malignancies, although they do contribute to a reduction in unnecessary FNAB procedures [8]. In contrast, the modified McGill score appears more promising for pediatric use, though current data are preliminary and require validation in larger, prospective cohorts [9,10].

Currently, the diagnostic approach to pediatric thyroid nodules largely mirrors that of adults. Nonetheless, the interpretation of findings and subsequent management decisions are often more complex in children, especially in cases of cytologically indeterminate nodules.

The internationally adopted Bethesda System for Reporting Thyroid Cytopathology (TBSRTC)—most recently updated in 2023—remains the standard classification system for cytological classification across age groups, including pediatric patients [11]. Key updates from the 2017 edition include elimination of the term follicular lesion of undetermined significance (FLUS), greater emphasis on nuclear atypia—associated with a higher risk of malignancy (ROM) within Category III—and removal of the term suspicious for a follicular neoplasm from Category IV. Indeterminate thyroid nodules fall into two key Bethesda categories:

- Category III (AUS): Atypia of undetermined significance, now further subclassified based on the presence or absence of nuclear atypia;
- Category IV (FN): Follicular neoplasm or suspicious for follicular neoplasm.

The 2023 revision reaffirmed the applicability of the Bethesda System in children, providing updated malignancy risk estimates and corresponding management strategies for each category. In pediatric populations, the ROM is reported to be approximately 28% for AUS (Category III) and 50% for FN (Category IV) nodules. Given these elevated risks, surgical intervention is typically recommended; however, repeat FNAB and close observation may be considered in AUS cases without nuclear atypia.

Management recommendations vary slightly among clinical guidelines. The 2022 European Thyroid Association (ETA) guidelines advocate for repeat FNAB after six months in Bethesda III and IV nodules. In contrast, the 2015 American Thyroid Association (ATA) guidelines suggest lobectomy and isthmectomy for most indeterminate lesions [5,6].

In Italy, the SIAPEC (Società Italiana di Anatomia Patologica e Citologia) classification system [12] is commonly used. This system stratifies indeterminate nodules into:

- TIR3A: Low-risk indeterminate lesions for which FNAB repetition after six months is recommended;
- TIR3B: High-risk indeterminate lesions, generally managed with lobectomy and isthmectomy.

The low-risk indeterminate category is characterized by low cellularity, a scarce colloidal component, and numerous microfollicular structures that are insufficient to define a follicular neoplasm. In contrast, the high-risk indeterminate is characterized by high cellularity and a monotonous arrangement in microfollicular structures with a very low or absent colloid, features suggestive of a follicular neoplasm.

Although the system does not differentiate between adult and pediatric cases, an Italian study confirmed its clinical applicability in the pediatric population [13]. This study demonstrated that TIR3A lesions may be safely managed with active surveillance, while TIR3B lesions require surgical intervention, aligning with adult management strategies.

A third classification system, established by the British Thyroid Association (BTA) [14,15], is also used in some centers. It categorizes indeterminate nodules as follows:

- Thy3a: Atypia of undetermined significance, managed with repeat FNAB;
- Thy3f: Follicular neoplasm or suspicious for follicular neoplasm, typically warranting lobectomy.

Given the higher malignancy risk in pediatric nodules, direct application of adult-based strategies may be inappropriate. Management remains heterogeneous, affected by factors such as the cytological system used, referral bias in tertiary centers (where more suspicious nodules concentrate), and surgeon experience [16].

In recent years, molecular analysis of FNAB samples has emerged as a promising tool to identify oncogenic mutations or gene fusions that may guide clinical decision-making. Detection of specific oncogenic mutations or gene fusions may help guide management, supporting either continued surveillance or surgical intervention (lobectomy or total thyroidectomy).

The choice of surgical approach should be carefully individualized and discussed with the patient and their family, balancing expected benefits with potential risks. Among possible complications, recurrent laryngeal nerve injury, though rare, can lead to vocal cord paralysis. The most common complication—especially after total thyroidectomy—is hypoparathyroidism, which can have long-term clinical implications [17].

To date, no systematic reviews have been published specifically addressing the surgical management of indeterminate thyroid nodules in the pediatric population. Therefore, the aim of this systematic review is to critically analyze and synthesize available data on the surgical management strategies, indications, and outcomes in children and adolescents with cytologically indeterminate thyroid nodules.

## 2. Materials and Methods

This systematic review and meta-analysis was conducted in accordance with the Preferred Reporting Items for Systematic Reviews and Meta-Analyses (PRISMA) guidelines. A comprehensive literature search was performed using the bibliographic databases MEDLINE and Embase, covering the period from January 2000 to June 2025. The search strategy included terms related to indeterminate thyroid nodules, surgical management, and malignancy rate in the pediatric population.

All retrieved citations, titles, and abstracts were imported into Rayyan, an online tool for systematic review management. Only original observational research articles published in English were considered for inclusion. To be eligible, studies had to report clearly defined numerical data on the malignancy rate and subsequent surgical management, including:

- The total number of cases;
- Malignancy rates stratified by indeterminate cytological category (e.g., Bethesda III and IV, TIR3A/B, Thy3a/f);
- Corresponding surgical strategies.

After removing duplicates, six medically trained experts in pediatric endocrinology and endocrine surgery (GT, JM, AB, FC, GC, LDS) independently screened all titles and abstracts to exclude clearly irrelevant articles. Full-text review of potentially relevant studies was then conducted independently reviewed by three experts (GT, FC, JM) to confirm inclusion eligibility based on predefined criteria.

Data extraction was carried out independently by two reviewers (GT, JM) for each included study. Extracted variables included:

- Author(s) and year of publication;
- Study location and setting;
- Population characteristics;
- Malignancy rate by cytological category;
- Recommended surgical management.

The quality of reporting for each included study was assessed independently by two reviewers (FC, LDS) using a checklist adapted from the STROBE (Strengthening the Reporting of Observational Studies in Epidemiology) guidelines, specifically tailored for observational studies in rare diseases. Each study received an overall rating of low, medium, or high quality based on five evaluation criteria:

1. Description of study design and setting;
2. Eligibility criteria;
3. Study population characteristics;
4. Reported outcomes;
5. Study participant flow and characteristics.

Disagreements in quality scoring were resolved by discussion or with the involvement of a third reviewer (GT).

Although ethical approval was not required for a systematic review, ethical considerations relevant to medical research were addressed, ensuring transparency, accuracy, and responsible synthesis of evidence. Particular attention was paid to the appropriate handling of sensitive data and the respectful citation of all original sources. The systematic review was not registered in a public database due to the highly specific nature of the topic.

## 3. Results

**Figure 1:**
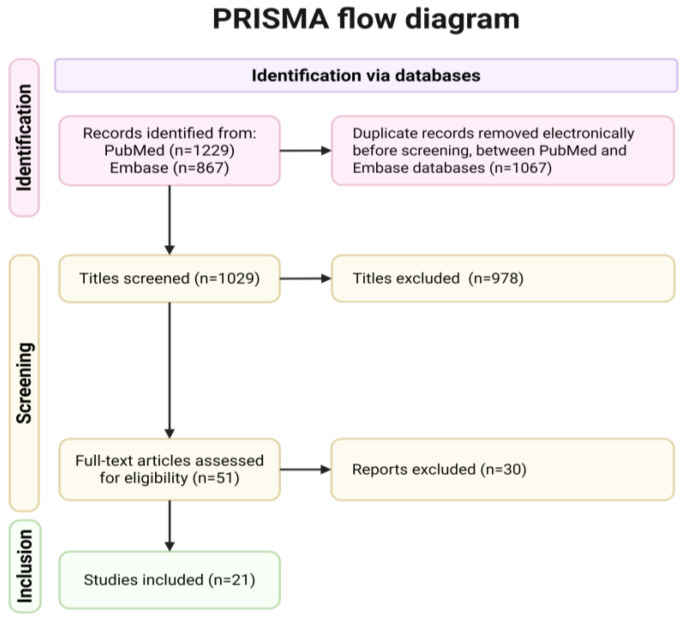
PRISMA flowchart showing the process of literature search and study selection.

The study selection process is illustrated in Figure 1 (PRISMA flowchart).

A total of 2096 records were identified through systematic searches of the MEDLINE and Embase databases. After removing 1067 duplicate entries, 1.029 titles and abstracts were screened for relevance. Of these, 978 records were excluded based on title and abstract review for not meeting the inclusion criteria. The remaining 51 full-text articles were assessed in detail for eligibility. Following full-text review, 21 studies (1% of total records) met all inclusion criteria and were included in the final systematic review.

The characteristics of the included studies are represented in Table 1 [10,13,16,18,19,20,21,22,23,24,25,26,27,28,29,30,31,32,33,34,35]. Most included studies were conducted in the USA (12/21, 57%) [10,19,20,23,25,26,27,28,29,30,33,35]. The remaining studies were conducted in Turkey (4/21, 19%) [21,24,31,34], Italy (1/21, 4.8%) [13], France (1/21, 4.8%) [22], Australia (1/21, 4.8%) [18], Portugal and Turkey (1/21, 4.8%) [31] and one was a meta-analysis (1/21, 4.8%) [16].

**Figure 2:**
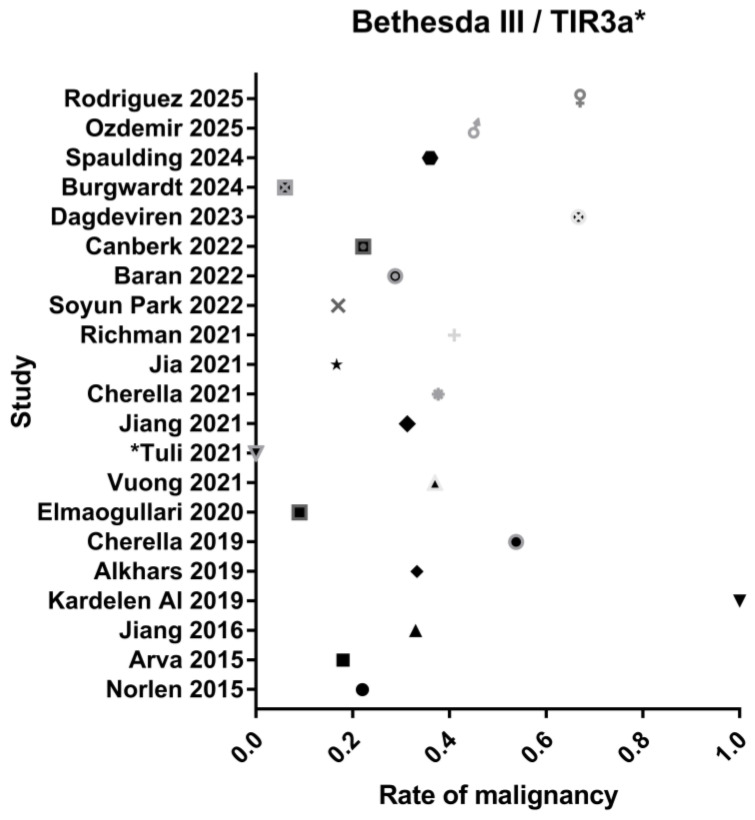
Rate of malignancy in the low-risk indeterminate category [18,19,20,21,22,23,24,25,26,27,28,29,30,31,32,33,34,35].

**Figure 3:**
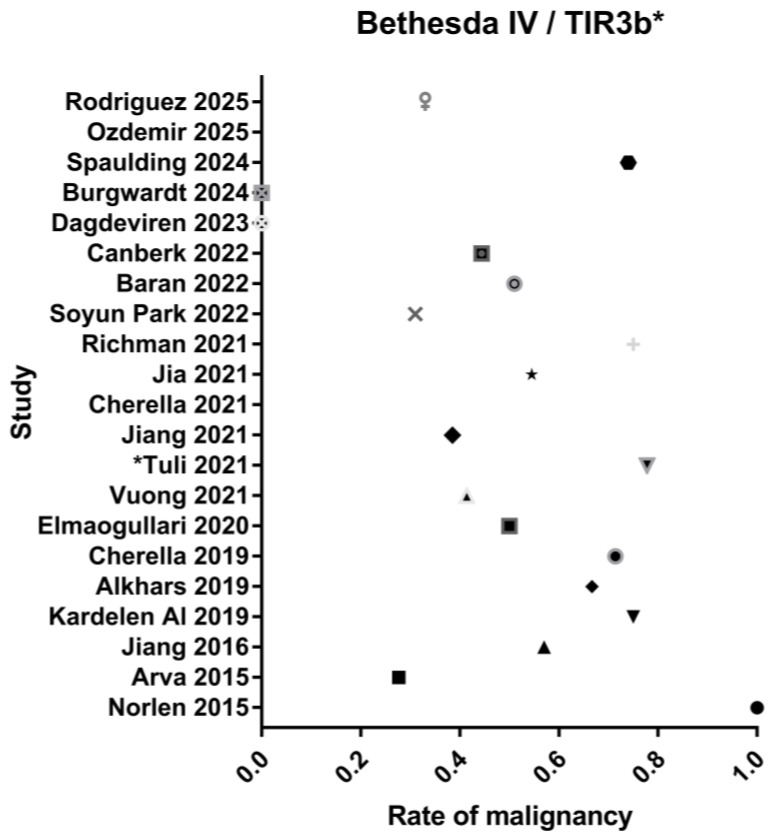
Rate of malignancy in the high-risk indeterminate category [18,19,20,21,22,23,24,25,26,27,28,29,30,31,32,33,34,35].

Malignancy rates for low-risk and high-risk indeterminate categories are represented in Figure 2 and Figure 3, respectively.

Most papers (9 out of 21, 42.9%) advocate for diagnostic lobectomy for indeterminate thyroid nodules, in accordance with the 2015 American Thyroid Association (ATA) guidelines [21,22,23,24,27,28,29,32,35]. TSH levels were correlated to malignancy, whereas previous history of radiotherapy, thyroid autoimmune disease, nodule ultrasound feature, gender, and age were not different [21]. Given the significant difference in malignancy between pediatric and adult age, a more interventistic approach was advocated for children and adolescents [23]. A gene-driven decision to perform lobectomy rather than total thyroidectomy, or clinical observation rather than lobectomy, is recommended in 2 out of 21 papers (9.5%) [25,33]. Repetition of fine-needle aspiration biopsy (FNAB) or surgical resection is suggested for nodules ≥ 4 cm in cases of Bethesda III, and resection is recommended for Bethesda IV in 2 out of 21 papers (9.5%) [18,19].

The presence of nuclear atypia is considered a justification for lobectomy in Bethesda III nodules, whereas FNAB repetition is indicated in the absence of nuclear atypia, according to 2 out of 21 papers (9.5%) [26,34]. One recent meta-analysis (1 out of 21, 4.8%) reports that the risk of malignancy (ROM) difference between Bethesda III and IV is not statistically significant and therefore does not warrant different management strategies [16].

In 4 out of 21 studies (19%), heterogeneous surgical approaches are reported: lobectomy for nodules ≥ 4 cm with possible total thyroidectomy; decision-making guided by a modified McGill Thyroid Nodule Score; direct surgery in some cases; lobectomy alone in the presence of low-risk oncogenic drivers; or lobectomy with central lymphadenectomy if high-risk oncogenic drivers are identified [10,20,30,31].

To date, only one study (1 out of 21, 4.8%) has addressed the Italian SIAPEC cytological classification, recommending FNAB repetition for TIR3a lesions and total thyroidectomy for TIR3b lesions due to the high associated ROM (77.8%) [13].

Risk factors for malignancy were evaluated in 11 of the 21 studies included (52.4%) [13,20,21,24,26,27,28,30,32,33,34]. Age at diagnosis was consistently not associated with malignancy (7/7 studies, 100%), whereas male sex was reported as a risk factor in six of eight studies (75%). A history of head and neck radiotherapy was not related to malignancy (4/4 studies, 100%). Concomitant autoimmune thyroid disease was associated with malignancy in one of four studies (25%). Elevated TSH levels were linked to malignancy in two of four studies (50%), and larger nodule size in four of eight studies (50%). Ultrasound features were predictive of malignancy in four of seven studies (57.1%), while a TIRADS score was associated with malignancy in one of two studies (50%).

Regarding molecular findings, mutations in *DICER1*, *PTEN*, *RAS*, and *TSHR* were classified as low-risk, whereas *BRAF* mutations or gene fusions were considered high-risk [30,33].

## 4. Discussion

Thyroid nodules are relatively uncommon in the pediatric population compared to adults; however, they carry a higher risk of malignancy (ROM), particularly in cases with cytologically indeterminate lesions or non-diagnostic samples [23]. TBSRTC, the most widely used framework for classifying thyroid FNA results, was updated in 2023 [11]. Notably, for the first time, the 2023 update includes specific management recommendations for pediatric patients. For Bethesda category III, the updated system recommends either repeat fine-needle aspiration (FNA) or surgical resection, while surgical resection is advised for category IV.

However, the most recent guidelines from the American Thyroid Association (ATA) and the European Thyroid Association (ETA) differ in their approach recommendations for Bethesda III and IV nodules. The ATA advises either repeat FNAB or lobectomy, whereas the ETA recommends repeat FNAB alone [5,6]. In our systematic review, 42.9% of included studies advocated for lobectomy in cases of indeterminate thyroid nodules [21,22,23,24,27,28,29,32,35]. This surgical approach may be justified by the high ROM observed in both Bethesda III and IV categories, with no statistically significant difference between them, calling into question the need for differing management strategies between the two, as confirmed in the meta-analysis of Vuong et al. [16]. However, a more nuanced approach may be warranted: the presence of nuclear atypia within Bethesda III nodules supports lobectomy, while its absence may justify a surveillance strategy with clinical and ultrasound follow-up may be more appropriate [26,34]. Additionally, genetic analysis of FNAB specimens, suggested in two of the included studies, offers further risk stratification by identifying oncogenic drivers [25,33]. Mutations such as *RAS*, *DICER1*, or *PTEN* are generally associated with lower-risk behavior, while *BRAF* mutations or kinase fusions suggest a higher risk for invasive disease [30,36]. Integrating molecular profiling into the diagnostic algorithm may therefore improve individualized management decisions in pediatric patients with indeterminate thyroid nodules.

Nodule size represents another important consideration in surgical decision-making. Several authors suggest lobectomy in the presence of nodules ≥ 4 cm, regardless of cytological category, given the higher likelihood of neoplastic transformation in larger lesions [18,20]. This size-based criterion may therefore serve as an additional determinant in managing borderline or indeterminate cases. However, the role of total thyroidectomy for indeterminate thyroid nodules in pediatric patients remains controversial. While this approach ensures complete removal of potentially malignant tissue and facilitates postoperative radioactive iodine therapy if needed, it also carries a higher risk of complications, including hypoparathyroidism and recurrent laryngeal nerve injury. Given these risks and the indeterminate nature of Bethesda III and IV categories, a more conservative approach—such as initial lobectomy—may be favored in many cases, reserving total thyroidectomy for confirmed malignancy or bilateral disease [21,22,23,24,27,28,29,32,35].

Variation in management approaches among tertiary care centers may be influenced by multiple institutional factors and potential biases. These include differences in the observed malignancy rates for indeterminate nodules at each center, referral bias stemming from the concentration of larger or more complex cases at high-volume centers, and the expertise and preferences of multidisciplinary teams. This variability underscores the need for individualized risk assessment and highlights the importance of standardized, evidence-based protocols, particularly in the pediatric population, where balancing oncologic safety with the minimization of surgical morbidity is critical.

Regarding the British Thyroid Association (BTA) classification, the Thy3a category encompasses atypia of undetermined significance comparable to Bethesda category III [14]. The 2022 BTA pediatric guidelines recommend repeat fine-needle aspiration biopsy (FNAB) for Thy3a lesions [15]. The Thy3f category includes follicular neoplasm or suspicious for follicular neoplasm, which generally warrants diagnostic lobectomy, mirroring the Bethesda IV approach. However, we did not identify any studies specifically reporting the risk of malignancy (ROM) for each BTA category in pediatric patients nor studies evaluating the subsequent clinical or surgical management decisions based on this classification. This highlights a current gap in the literature and underscores the need for pediatric-specific validation of the BTA system.

The SIAPEC classification, adopted in Italy since 2014, differs notably from the Bethesda and BTA systems [12]. While this stratification aims to better guide clinical management—typically favoring surveillance for TIR3a and lobectomy for TIR3b—there is currently a lack of pediatric-specific data evaluating ROM and outcomes based on the SIAPEC system. To date, only one published study has addressed the SIAPEC classification in the pediatric population, involving a small monocentric cohort [13]. In that study, FNAB repetition was advocated for TIR3a cases due to the absence of malignancy on follow-up, except when the nodule measured ≥ 4 cm, in which case surgical intervention was considered. For TIR3b nodules, total thyroidectomy was recommended, based on a high reported ROM of 77.8%, supporting the classification of this category as high-risk. Despite these findings, further multicenter studies with larger pediatric cohorts are needed to validate the applicability and predictive value of the SIAPEC system in children.

The strength of this study is the systematic approach and the extensive review of the literature, which was critically evaluated by experts managing pediatric thyroid nodules in a tertiary institute. The limits of this study are the retrospective nature of most studies and the heterogeneity of the clinical and surgical management among the tertiary institutes that did not allow the meta-analysis.

Future research directions should include a standardized approach to the risk factors assessment for thyroid nodule malignancy as well as to the clinical and surgical management.

A suggested approach to improve consistency and accuracy in the management of indeterminate thyroid nodules in pediatric patients is the development of an internationally accepted risk stratification score specifically tailored to the pediatric population. This tool should integrate multiple relevant domains to provide a comprehensive, individualized risk assessment, including:

- Clinical factors: age, gender, underlying thyroid disease (e.g., autoimmune thyroiditis, primary hypothyroidism), history of radiation exposure, prior oncologic treatment, and family history of thyroid or other endocrine malignancies;
- Ultrasound characteristics: nodule echogenicity, intranodular calcifications, vascularization patterns, nodule localization, and the presence of suspicious cervical lymphadenopathy;
- Cytological classification: based on standardized systems such as Bethesda, BTA, or SIAPEC;
- Molecular profile: identification of oncogenic drivers with prognostic significance, distinguishing between low-risk mutations (e.g., *RAS*, *DICER1*, *PTEN*) and high-risk alterations (e.g., *BRAF*, *RET/PTC* fusions, or other kinase rearrangements).

Such a comprehensive scoring system could support clinical decision-making by helping stratify patients into appropriate management pathways, ranging from observation and repeat FNAB to lobectomy or total thyroidectomy. Ultimately, this approach aims to optimize oncologic outcomes while minimizing overtreatment and surgical morbidity. Crucially, the proposed model should undergo prospective validation through multicenter studies to ensure its applicability, reliability, and generalizability across diverse pediatric populations.

## 5. Conclusions

The rarity of thyroid nodules in the pediatric population, combined with the heterogeneous management strategies adopted by the various tertiary centers included in this systematic review, limited the feasibility of conducting a meta-analysis focused on surgical approaches. Variability in institutional protocols, cytological classification systems, and criteria for surgery—including differences in size thresholds, use of molecular testing, and interpretation of indeterminate categories—further contributed to the lack of uniform data. As a result, while descriptive insights could be drawn, a pooled quantitative analysis comparing outcomes by surgical strategy was not possible. This highlights the urgent need for standardized pediatric-specific guidelines and multicenter collaborative studies to inform evidence-based management of indeterminate thyroid nodules in children.

## Figures and Tables

**Table 1:** Surgical indications in the included studies.

Author	Year	Patients *	Histopathological Rate of Malignancy	Overall Rate of Malignancy	Surgical Indications
Norlén [18] Australia	2015	15	22% Bethesda III, 100% Bethesda IV	20% Bethesda III, 100% Bethesda IV	Repeat FNAB or very close monitoring for Bethesda III and resection if ≥4 cm, surgical resection for Bethesda IV.
Arva [19] USA	2015	34	18.7% Bethesda III, 27.7% Bethesda IV	11.5% Bethesda III, 27.% Bethesda IV	Repeat FNAB or very close monitoring for Bethesda III, surgical resection for Bethesda IV.
Jiang [20] USA	2016	10	33% Bethesda III, 57% Bethesda IV	-	Indeterminate nodules ≥4 cm at a minimum lobectomy, perhaps not unreasonable to consider total thyroidectomy.
Kardelen Al [21] Turkey	2019	18	100% Bethesda III, 75% Bethesda IV	41.7% Bethesda III, 75% Bethesda IV	Lobectomy for indeterminate cytopathology.
Alkhars [22] France	2019	6	33.3% Bethesda III, 66.7% Bethesda IV	-	No indication for Bethesda III, diagnostic surgery for Bethesda IV.
Cherella [23] USA	2019	46	53.8% Bethesda III, 71.4% Bethesda IV	43.8% Bethesda III, 71.4% Bethesda IV	Lobectomy for atypia of unknown significance and follicular neoplasm.
Elmaoğullari [24] Turkey	2020	16	9% Bethesda III, 50% Bethesda IV	-	Lobectomy for indeterminate cytological category.
Vuong [16]	2021	Meta analysis	-	37% Bethesda III, 41.4% Bethesda IV	No significant difference between Bethesda III and IV to advocate different management. Pediatric Bethesda V should not be considered as indeterminate.
Tuli [13] Italy	2021	23	0% Bethesda III, 77.8% Bethesda IV	0% Bethesda III, 77.8% Bethesda IV	Observation for TIR3a and total thyroidectomy for TIR3b.
Jiang [25] USA	2021	36	31.3% Bethesda III, 38.5% Bethesda IV	22.7% Bethesda III, 35.7% Bethesda IV	Lobectomy for AUS and FN. Gene sequencing “rule in” test to determine total thyroidectomy rather than lobectomy.
Cherella [26] USA	2021	61	37.7% Bethesda III	35% Bethesda III	Lobectomy only on cases with nuclear atypia, FNAB repetition if other atypia.
Jia [27] USA	2021	78	16.7% Bethesda III, 54.5% Bethesda IV	15.6% Bethesda III, 54.5% Bethesda IV	Lobectomy or repeat FNAB in Bethesda III. Surgical resection in Bethesda IV.
Richman [28] USA	2021	78	41% Bethesda III, 75% Bethesda IV		Diagnostic lobectomy for Bethesda III and IV.
Soyun Park [29] USA	2022	43	17% Bethesda III, 31% Bethesda IV	-	Surgical removal for indeterminate category.
Baran [30] USA	2022	126	28.8% Bethesda III, 51% Bethesda IV	21.7% Bethesda III, 51% Bethesda IV	Lobectomy for AUS and FN and a driver oncogenic alteration associated with low invasive risk. Lobectomy with prophylactic neck dissection or AUS and FN and a driver mutation associated with high invasive risk.
Canberk [31] Portugal + Turkey	2022	76	22.2% Bethesda III, 44.4% Bethesda IV	10% Bethesda III, 33.3% Bethesda IV	Direct surgery for AUS is questionable.
Dağdeviren Çakir [32] Turkey	2023	5	66.7% Bethesda III, 0% Bethesda IV	-	Lobectomy for AUS, no definite conclusion for FN due to very few cases.
Burgwardt [10] USA	2024	17	6% Bethesda III, 0% Bethesda IV	-	AUS with Modified McGill Thyroid Nodule Score: <10 observation, >10 and <12 lobectomy, ≥12 total thyroidectomy.
Spaulding [33] USA	2024	41	36% Bethesda III, 74% Bethesda IV	-	Surgery rather than FNAB repetition in case of *BRAF*, *NRAS*, or *DICER1* mutation.
Ozdemir Uslu [34] Turkey	2025	20	45% Bethesda III	32.1% Bethesda III	Lobectomy only for nuclear atypia, FNAB repetition in case of other atypia.
Rodriguez [35] USA	2025	19	67% Bethesda III, 33% Bethesda IV	30.8% Bethesda III, 16.7% Bethesda IV	Lobectomy for AUS and FN.

* Only patients with cytologically indeterminate nodules were included in the analysis. AUS: Atypia of Unknown Significance; FN: Follicular Neoplasm.

## Data Availability

All data are available upon request to the corresponding author.

## Abbreviations

The following abbreviations are used in this manuscript:

ROM	Rate of Malignancy
FNAB	Fine Needle Ago Biopsy
TIRADS	Thyroid Imaging Reporting and Data System
TBSRTC	The Bethesda System for Reporting Thyroid Cytopathology
AUS	Atypia of Undetermined Significance
FN	Follicular Neoplasm
ETA	European Thyroid Association
ATA	American Thyroid Association
SIAPEC	Società Italiana di Anatomia Patologica e Citologia
BTA	British Thyroid Association
PRISMA	Preferred Reporting Items for Systematic Reviews and Meta-analyses
STROBE	Strengthening the Reporting of Observational Studies in Epidemiology
FLUS	Follicular Lesion of Undetermined Significance
